# 

**Published:** 1898-10-29

**Authors:** 


**Notices of Births, Deaths, and Marriages** are inserted in this column at a charge of 2s. 6d. for an announcement not exceeding four lines.

### NOTICE TO CORRESPONDENTS.

All MS., Letters, Books for Review, and other matters intended for the Editor should be addressed The Editor, "The Hospital," The Hospital Building, 28 & 29, Southampton Street, Strand, W.C.

The Editor cannot undertake to return rejected MS., even when accompanied by stamped directed envelope.

**Notice to Advertisers and Subscribers.**

All Advertisements, Orders for Copies of The Hospital, and Business Communications should be addressed to **THE MANAGER (Not to the Editor),** The Hospital, The Hospital Building, 28 & 29, Southampton Street, Strand, London, W.C.

The Cost of Subscription, post free, is as follows:—Per week, 2½d.; per quarter, 2s. 8d.; per half-year, 5s. 3d.; per annum, 10s. 6d. Foreign:—Per annum, 15s. 2d., payable, in all cases, in advance.

**NOTICE.—Vol. XXIV. of "The Hospital" (April, 1898, to September, 1898), in handsome cloth binding, is now on sale at the Office of this Journal, price 7s. 6d. Cases for binding the Numbers contained in this Volume 1s. 6d. Index to Vol. XXIV., 2½d., post free.**

**The Monthly Part for November will be ready November 1st. Price 6d., by post 8d.**

### A GENEROUS GIFT.

ANCOATS HOSPITAL at Manchester has for some time past been in great need of a new out-patients department. Through the generosity of the sons and daughter of the late hon. treasurer, Mr. James Oliver, this want is to be supplied, free of cost to the hospital. The expenditure is estimated at from £6,000 to £7,000.

### BOOKS RECEIVED.

The Scientific Press.

"An Atlas of Bacteriology." By Charles Slater, M.A., M.B., M.R.C.S.Eng., and Edmund J. Spitta, L.R.C.P.Lond., M.R.C.S.Eng.

The Sanitary Publishing Co.

"Disinfection and Disinfectants." By Samuel Rideal, D.Sc.Lond. Second Edition.

Isbister and Co.

"Bishop Walsham How." By Frederick Douglas How. With portrait.
"The Laurel Walk." By Mrs. Molesworth.

S. W. Partridge and Co.

"The Zenana." Vol. V. 1897-8.

L. Carriere, Alexandria.

"Abscès du Foie." By Dr. A. P. Petridis.

### Scraps and Gleanings.

The cost per head of ordinary patients at the Buluwayo Hospital is 8s. 8d. per day.

An anonymous donor has presented the Sheffield Royal Hospital with Sheffield Corporation Water Annuities, representing a capital value of £19,443 odd.

The York City Council have applied to the Local Government Board for sanction to borrow £2,500 for the purchase and necessary furnishings of the Muncaster House Estate for hospital purposes.

Some 1,200 to 1,500 patients per month are treated in the out-patient department of the Birmingham Homœopathic Hospital. When the extensions in contemplation are completed the accommodation for in-patients will be 50 beds.

A benefit trip of the well known yacht "Skylark" took place off Brighton on October 3rd in aid of the Sussex County Hospital. This is the twenty-ninth annual benefit organised by the captain of the yacht, and this one was as great a success as ever.

At a recent meeting of the Dundee District Committee of the Forfar County Council the proposal to erect a fever hospital for the Dundee and Forfar districts, providing accommodation for 24 beds and costing about £5,000, was unanimously agreed to; and the committee were granted power to obtain plans and specifications of the proposed hospital.

Since the establishment of the Bradford Joint Hospital Fund in 1873 a total of nearly £18,000 has been collected. This amount, however, in the official view, is not considered as adequately representing year by year the interest of the workpeople of the city in these great charities, seeing that Bradford's contributions only equal about 2d. per head, while Leeds contributions equal about 4½d.; Birmingham, about 8d.; and Wigan about 1s. 4½d. per head.

At the Southampton Free Eye and Ear Hospital the quaint custom of having an annual "Pound" Day is in vogue, when visitors to the institution are invited to bring gifts of various articles, each weighing one pound.

The Birmingham Hospital Sunday Collection this year is to be in aid of the General Hospital. Out of the annual expenditure of this institution of £22,000, some £12,000 has to be raised by donations, congregational collections, &c.

Thirty-six Thousand Five Hundred and Seventy-two out-patients attended at the Birmingham General Hospital during the first eight months of 1898, while the total number of in-patients for the same period was 2,705.

The Duke of Norfolk (president of the Centenary Fund of the Sheffield Royal Infirmary) has issued an appeal for a further sum of £10,000, which is to be expended on the erection of a suitable operating theatre and the enlargement of the present out-patient rooms.

The result of the Aberdeen Hospital Saturday Fund movement for this year was £1,231 odd. There was a large increase over previous years in the sum raised at the annual sports and demonstration, but a slight falling off is shown in the workshop and business house collections.

The Local Government Board have received through the Foreign Office a communication from the Russian Chargé d'Affaires, stating that the Empress of Russia has expressed a desire to obtain photographs and plans from certain Poor Law institutions. The Local Government Board have mentioned the Ipswich Infirmary as the best typical institution of its kind, and the architect, Mr. H. Percy Adams, F.R.I.B.A., is engaged in preparing the plans for her Imperial Majesty. Mr. Adams is also the architect of the Bedford County Hospital.

### The Student of Medicine.

### Appointments.

A. J. Bennetts, M.R.C.S.Eng., L.R.C.P.Lond., appointed Assistant Medical Officer to the Lambeth Infirmary. A. C. Coles, M.D., B.Sc. Public Health (Edin.), appointed Medical Officer of Health to the Urban District Council, Winton, Bournemouth. E. Cuffey, M.B., R.U.I., appointed Surgeon to the Lady Strangford Hospital, Port Said. A. N. Davis, L.R.C.P., L.R.C.S.Edin., appointed Medical Superintendent to the Devonshire County Asylum, Exminster. R. B. Eskrigge, L.R.C.P.Lond., M.R.C.S.-Eng., appointed Medical Officer of Health to the Royston Urban District Council. J. Eyre, M.S., M.D.Durh., M.R.C.S., L.R.C.P., appointed Honorary Ophthalmic Surgeon to St. Mary's Children's Hospital, Plaistow. E. C. Goodbody, M.B.Dub., appointed District Medical Officer to the Dunmow Union. Dr. T. Jones, appointed Medical Officer for the Amlwch District of the Anglesey Union. Dr. T. O. Jones, appointed Medical Officer of Health to the Ruthin Rural District Council. J. Scott, M.B., C.M., appointed Medical Officer to the Indo-European Telegraph Company. T. P. G. Wells, L.R.C.P., L.R.C.S.Edin., appointed District Medical Officer to the St. Albans Union. A. T. Wilson, L.F.P.S.Glasg., appointed District Medical Officer to the Alcester Union.

